# Supplementary material for: A plant-like mechanism coupling m6A reading to polyadenylation safeguards transcriptome integrity and developmental gene partitioning in Toxoplasma
Source: eLife. 2021 Jul 15;10:e68312. doi: 10.7554/eLife.68312 (PMC8313237; doi:10.7554/eLife.68312)

RHKU80 METTL14-HAFLAG METTL3-(Ty)2

α FLAG → E1 E2 → SUP6

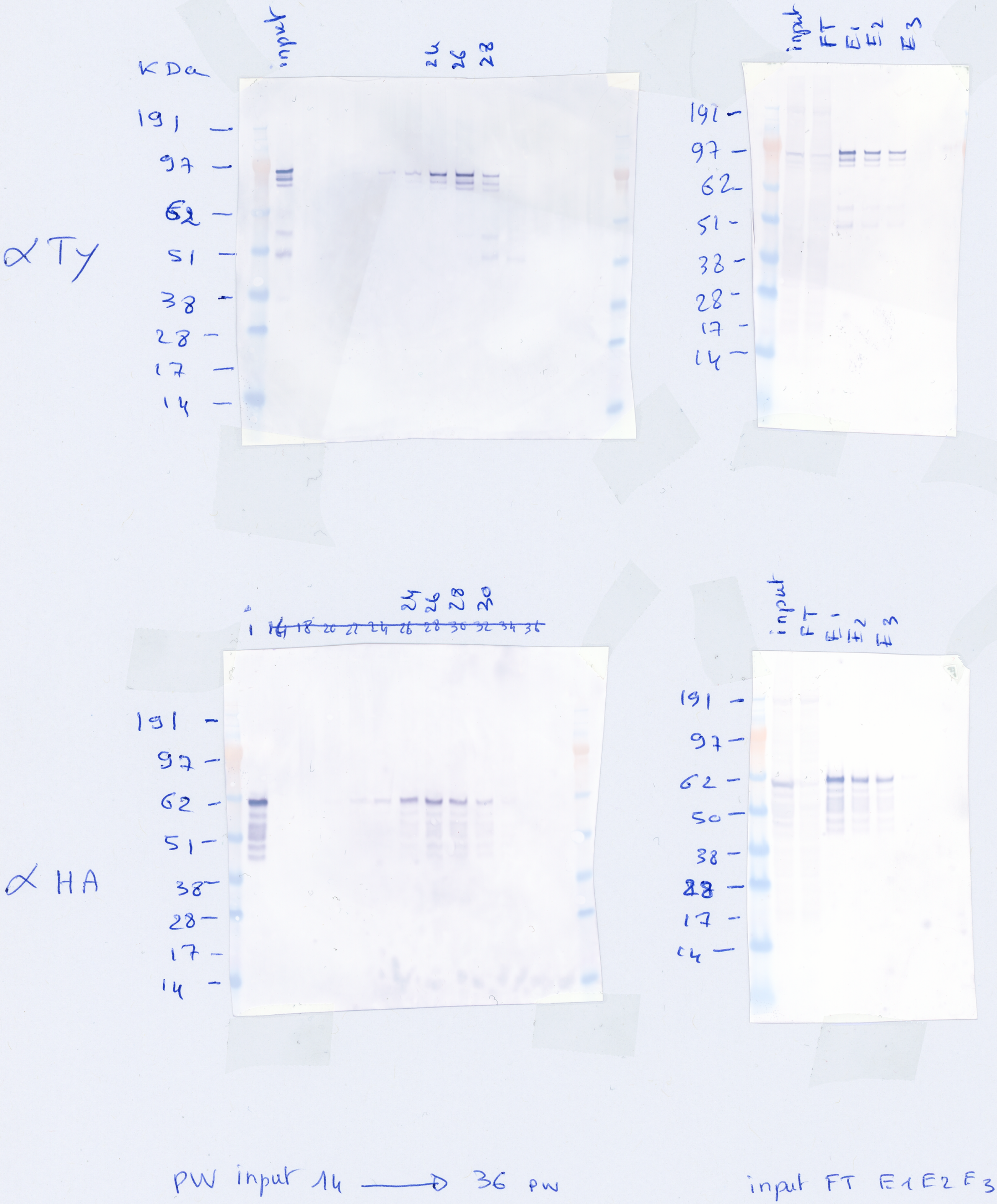

Supplement: Figure 2—source data 1. — Size markers (kDa) are indicated. [file elife-68312-fig2-data1.pdf]
