# Supplementary material for: A plant-like mechanism coupling m6A reading to polyadenylation safeguards transcriptome integrity and developmental gene partitioning in Toxoplasma
Source: eLife. 2021 Jul 15;10:e68312. doi: 10.7554/eLife.68312 (PMC8313237; doi:10.7554/eLife.68312)
Supplement: Figure 2—figure supplement 1—source data 1. — Size markers (kDa) are indicated. [file elife-68312-fig2-figsupp1-data1.pdf]

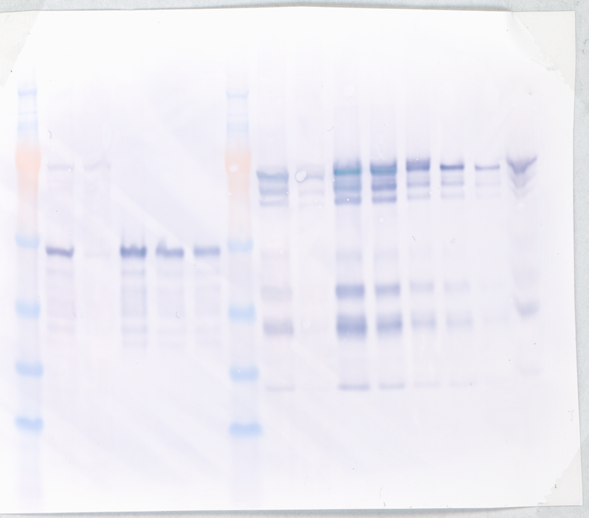

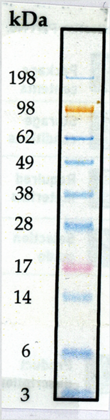

15. 1. 2021.

- purification
  MetL14 (38+180)
  et MetL3 (Sumofect)
- (MetL14 :
  - 50 p̄
    15 bleu
    5 DTT
  - fractions $E_1 - E_2 - E_3$
    E_3 : BC 100)
- MetL3 :
  65 p̄
  25 bleu
  10 DTT
  $E_1 E_2 E_3$ 650 peptide flag
  E_4 BC 500
  E_5 glycine.
- MES.
- Transfert 1h:30
- α-HA Mouse.

Rabbit
αHA
(cell signaling)

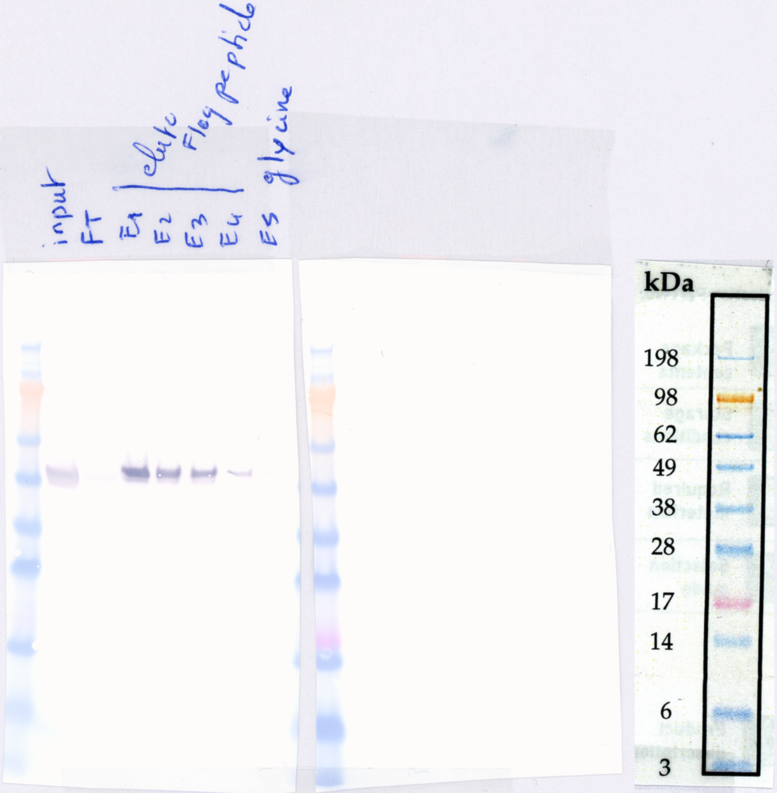

50 T180

RHKU80 WTAP-HAFlag

↓ Lysis Buffer Taxo

αIP FLAG

↓

Eluto. peptide
