# Supplementary figures and images for: A plant-like mechanism coupling m6A reading to polyadenylation safeguards transcriptome integrity and developmental gene partitioning in Toxoplasma
Source: eLife. 2021 Jul 15;10:e68312. doi: 10.7554/eLife.68312 (PMC8313237; doi:10.7554/eLife.68312)

METTL3<sup>maid</sup> HA

UT      IAA  
 5      8      24      28      Washout

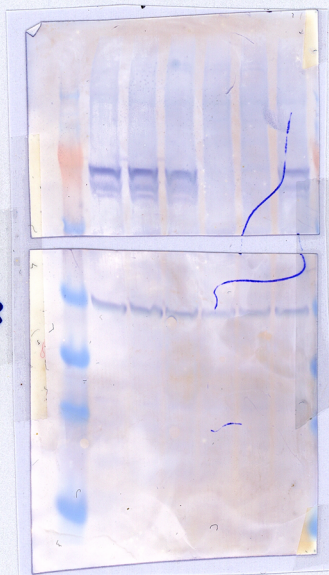

α HA

α HDAC<sub>3</sub>

kDa

198

98

62

49

38

28

17

14

6

3

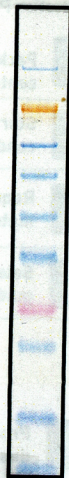

Supplement: Figure 3—source data 1. — Size markers (kDa) are indicated. [file elife-68312-fig3-data1.pdf]

CPS F30 MHA HA

T  
 0 5 8 24 48  
 IAA  
 without

$\alpha$ HA

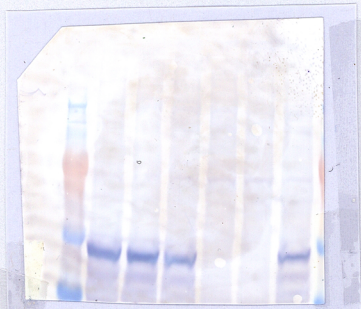

$\alpha$ ENO2

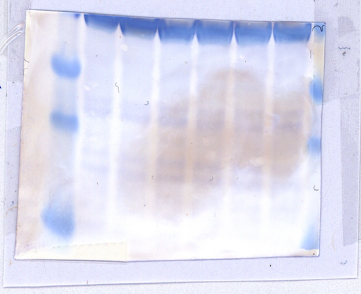

UT 4 8 24 48 W

$\alpha$ MIC2

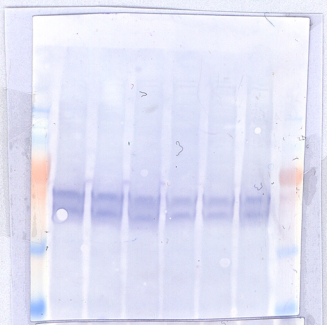

$\alpha$ GRA1

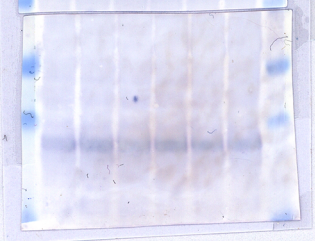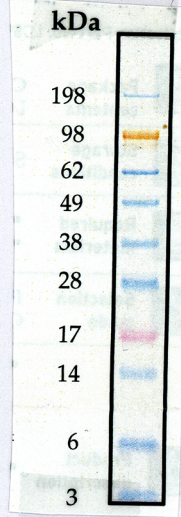

Supplement: Figure 7—source data 1. — Size markers (kDa) are indicated. [file elife-68312-fig7-data1.pdf]
