# Supplementary material for: A plant-like mechanism coupling m6A reading to polyadenylation safeguards transcriptome integrity and developmental gene partitioning in Toxoplasma
Source: eLife. 2021 Jul 15;10:e68312. doi: 10.7554/eLife.68312 (PMC8313237; doi:10.7554/eLife.68312)
Supplement: Supplementary file 2. [file elife-68312-supp2.pdf]

Crystal condition	Apo CPSF4YTH	CPSF4YTH / m6A	CPSF4YTH / m6A 7 mer RNA
**Beamline**	i04 (Diamond Light Source)	ID30-A1 (ESRF)	i04 (Diamond Light Source)
**Wavelength**	0.9795	0.9660	0.9795
**Resolution range**	34.53 - 1.23 (1.274 - 1.23)	30.76 - 1.349 (1.397 - 1.349)	31.04 - 1.38 (1.45 - 1.38)
**Space group**	P 41 21 2	P 1	P 1
**Unit cell**	40.18 40.18 202.6 90 90 90	32.47 35.22 38.18 114.51 100.44 97.77	32.44 35.15 38.35 114.68 100.70 97.65
**Total reflections**	544909 (14247)	49449 (3627)	65418 (506)
**Unique reflections**	49444 (4552)	30036 (2887)	19181 (288)
**Multiplicity**	11.0 (4.3)	1.6 (1.7)	3.4 (1.8)
**Completeness (%)**	99.32 (93.70)	92.64 (88.38)	62.86 (6.5)
**Mean I/sigma(I)**	20.3 (1.0)	6.6 (2.1)	17.3 (3.1)
**Wilson B-factor**	16.70	9.18	10.99
**R-merge**	0.045 (1.000)	0.092 (0.338)	0.025 (0.123)
**CC1/2**	1.0 (0.427)	0.956 (0.496)	0.999 (0.989)
**Reflections used in refinement**	49441 (4550)	30007 (2876)	19180 (153)
**Reflections used for R-free**	2500 (232)	1532 (144)	1918 (16)
**R-work**	0.1769 (0.3296)	0.1825 (0.2413)	0.1430 (0.1611)
**R-free**	0.2034 (0.3267)	0.1966 (0.2556)	0.1684 (0.1854)
**Number of non-hydrogen atoms**	1447	1408	1324
**macromolecules**	1293	1237	1232
**ligands**	4	23	31
**solvent**	150	148	61
**Protein residues**	158	150	149
**RMS(bonds)**	0.005	0.005	0.005
**RMS(angles)**	0.77	0.82	0.76
**Ramachandran favored (%)**	99.35	99.32	98.64
**Ramachandran allowed (%)**	0.65	0.68	1.36
**Ramachandran outliers (%)**	0.00	0.00	0.00
**Rotamer outliers (%)**	0.00	0.72	1.49
**Clashscore**	3.47	3.19	2.01
**Average B-factor**	22.56	13.21	14.72
**macromolecules**	21.50	11.61	14.11
**ligands**	26.98	17.20	20.91
**solvent**	31.58	25.93	23.89
**PDB id**	**7NG2**	**7NH2**	**7NJC**
