# Supplementary material for: A plant-like mechanism coupling m6A reading to polyadenylation safeguards transcriptome integrity and developmental gene partitioning in Toxoplasma
Source: eLife. 2021 Jul 15;10:e68312. doi: 10.7554/eLife.68312 (PMC8313237; doi:10.7554/eLife.68312)
Supplement: Supplementary file 4. [file elife-68312-supp4.pdf]

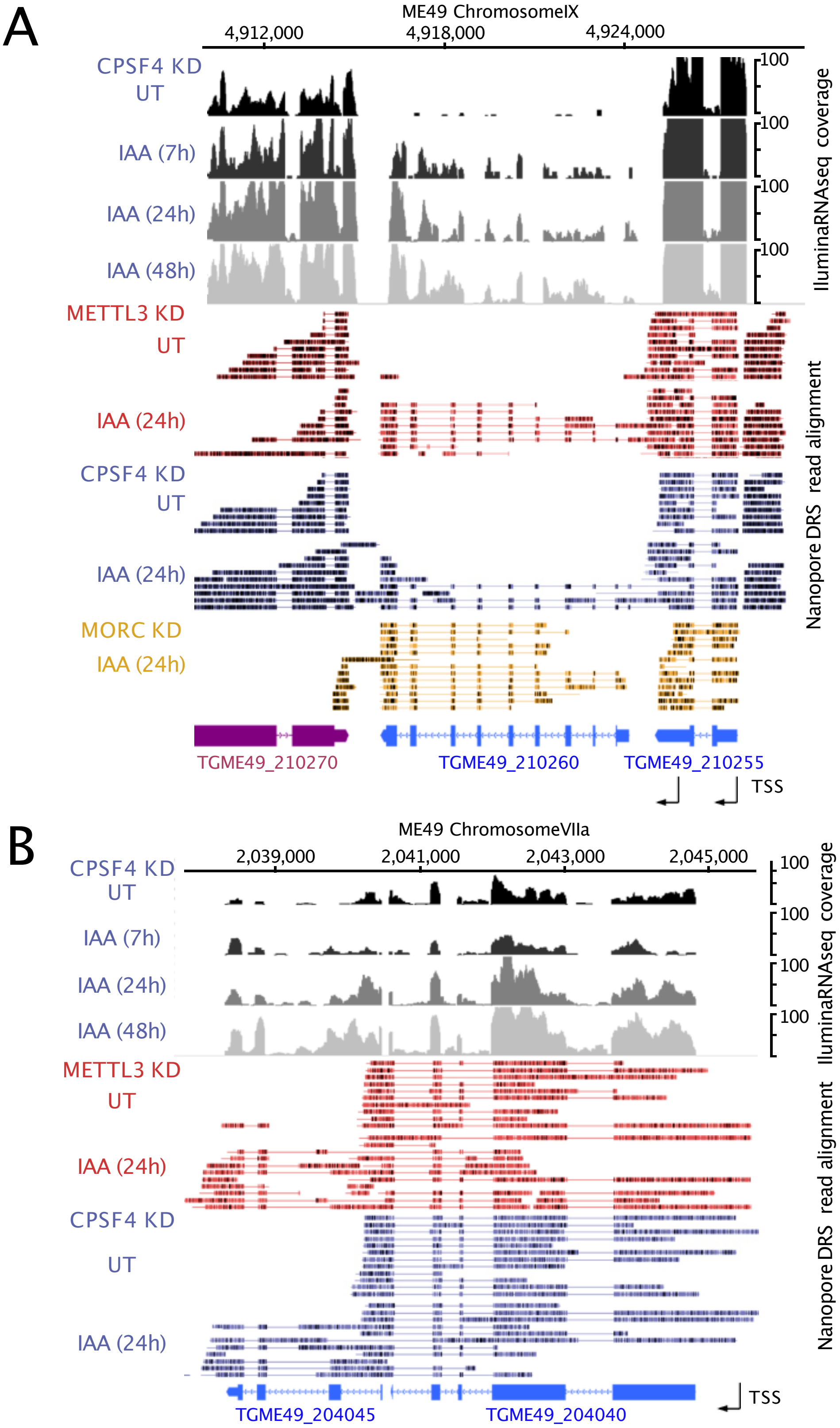

Read-through events and chimeric RNAs detected on T. gondii chromosomes VIIa and IX following CPSF4 and METTL3 post-translational knock-down. (**A**) and (**B**) illustrate representative examples of analysis of read-through events detected at different ***loci***. The chromosomal positions and gene accession numbers are shown for each panel. On top are displayed the ***illumina***-RNA-seq data before and after the IAA-dependent KD of CPSF4, at different times. The y-axis represents the RPKM values. Below is the nanopore-based DRS of aligned and sorted RNAs extracted before and after the IAA-dependent knock-down of CPSF4, METTL3 and MORC. Exons are shown with colored thick bars and introns with black lines, strand specific coloring is not shown. Notable observations: in (**A**), The respective nanopore data allows a clear assessment of the read-through/RNA chimera formation phenotype that is seen following the KD of both CPSF4 and METTL3 but not of MORC which only resulted in a conventional upregulation of the initially repressed gene ***TGME49_210260***. In (**B**), we can witness RNA chimera formation between ***TGME49_204040*** and ***TGME49_204045***.

Supplementary file 4

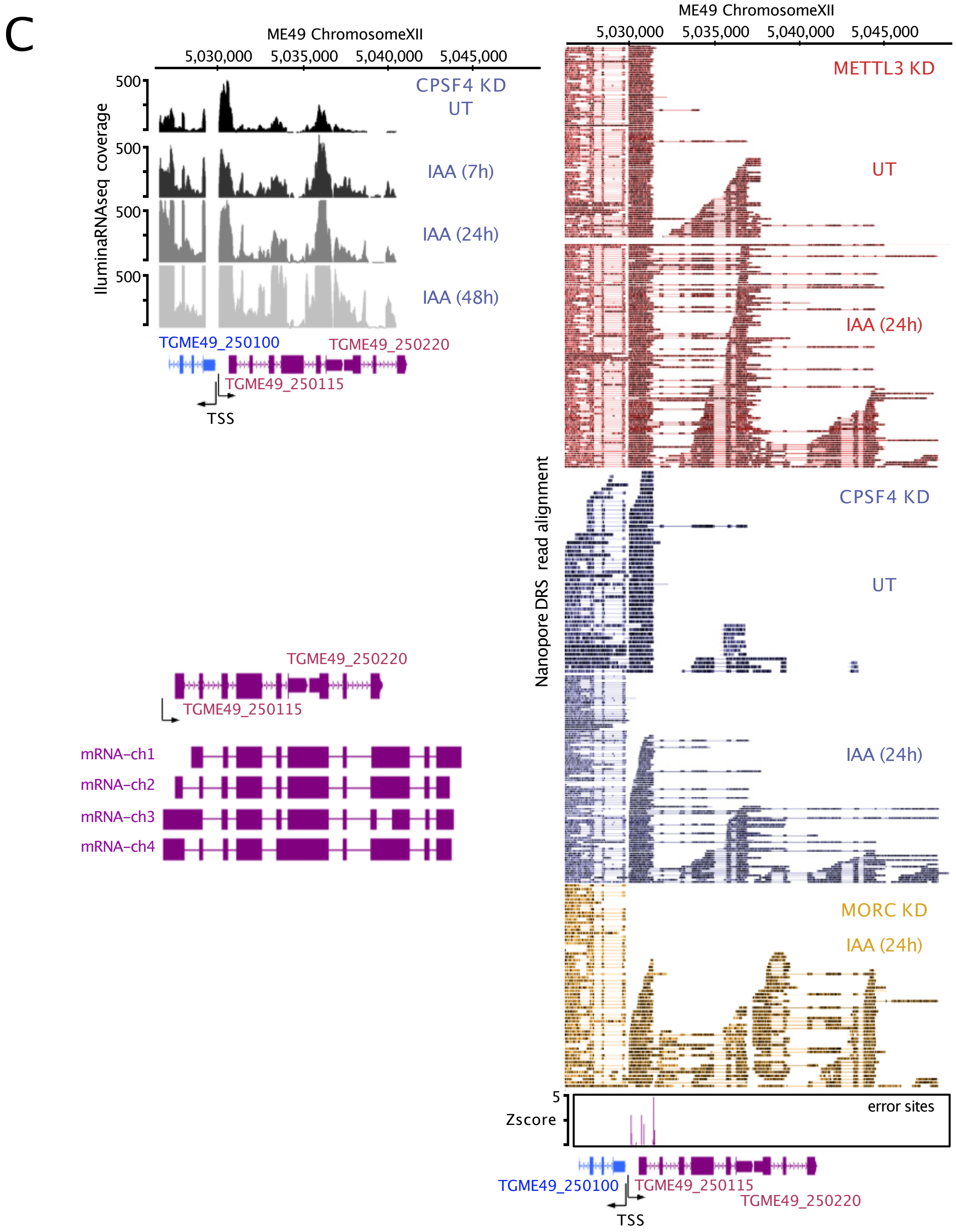

Read-through events and chimeric RNAs detected near TGME49_250115 locus following CPSF4 and METTL3 post-translational knock-down. A representative analysis of the read-through from the ***TGME49_250115*** into the ***TGME49_250220*** locus. (**top left panel**) Density profile from Illumina RNA-seq data before and after the IAA-dependent KD of CPSF4, at different time points. The y-axis represents the RPKM values. (**right panel**) Aligned Nanopore DRS reads of RNAs extracted before and after the IAA-dependent knock-down of METTL3 (in red), of CPSF4 (in blue) and of MORC (in yellow). Exons are shown with colored thick bars and introns or soft clipping with thinner lines, strand specific coloring is not used in this figure. The respective DRS data allows a clear assessment of the read-through phenotype that is seen following the KD of both CPSF4 and METTL3 but not of MORC which only resulted in a conventional upregulation of the initially repressed ***TGME49_250220*** gene and a clearly separated intergenic regions between TGME49_250115 and TGME49_250220. On the bottom is a histogram representation of ***Epinano*** differential error Z-score between METTL3 UT and IAA conditions. (**bottom left panel**) FLAIR derived read-through and splicing isoforms into the ***TGME49_250220 locus*** are displayed, as well as the differential splicing outcomes on the respective exons, resulting in chimeric RNAs. <u>Notable observations</u>: The data from the MORC KD in B allows to establish that the reads elongating beyond the annotated gene ***TGME49_250220***, correspond to a mis-annotated genomic sequence.

Supplementary file 4

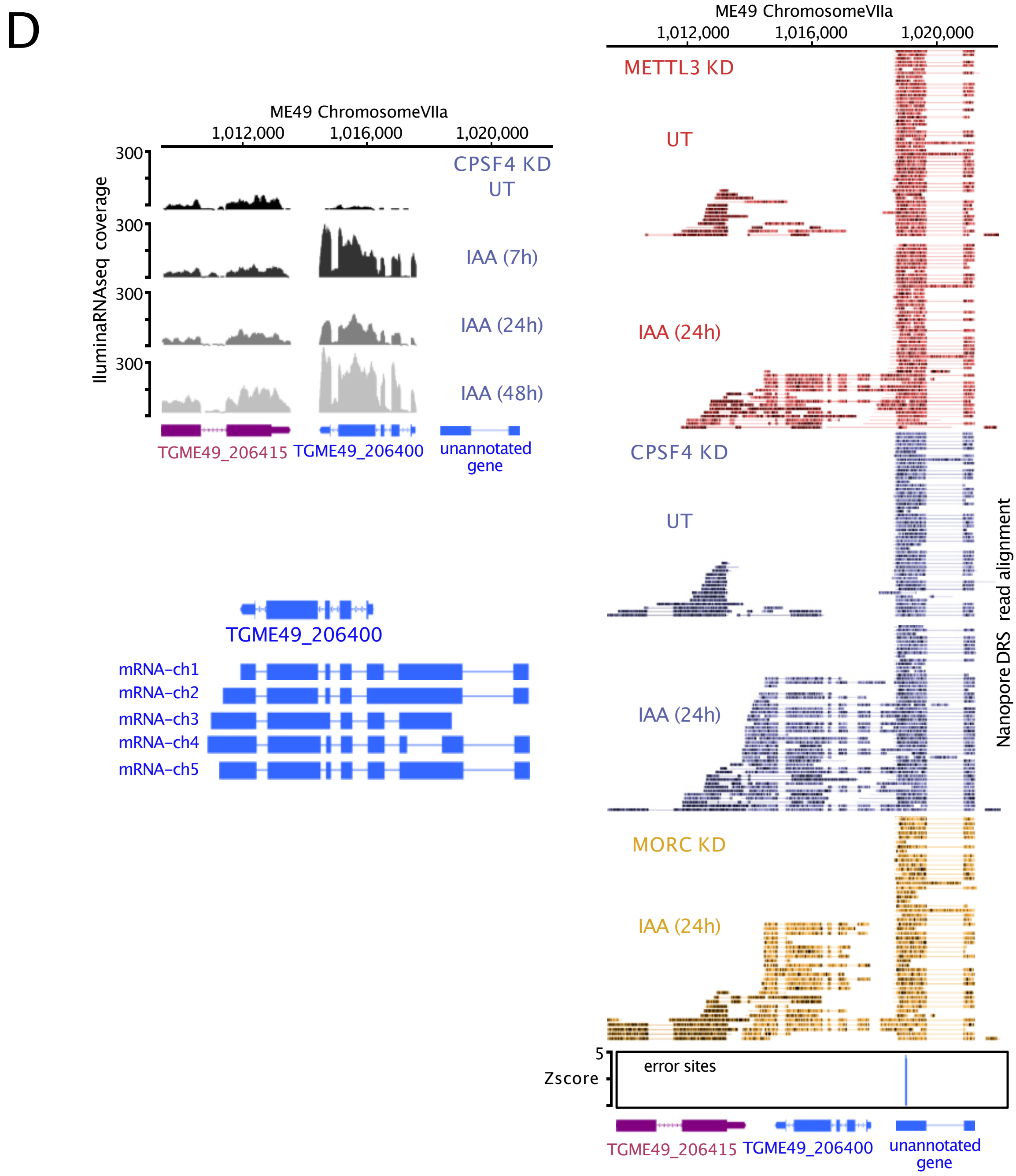

Read-through events and chimeric RNAs detected near TGME49_206400 locus following CPSF4 and METTL3 post-translational knock-down. A representative analysis of the read-through from an un-annotated gene into the ***TGME49_206400*** locus. (**top left panel**) Density profile from Illumina RNA-seq data before and after the IAA-dependent KD of CPSF4, at different time points. The y-axis represents the RPKM values. (**right panel**) Aligned Nanopore DRS reads of RNAs extracted before and after the IAA-dependent knock-down of METTL3 (in red), of CPSF4 (in blue) and of MORC (in yellow). Exons are shown with colored thick bars and introns or soft clipping with thinner lines, strand specific coloring is not used in this figure. The respective DRS data allows a clear assessment of the read-through phenotype that is seen following the KD of both CPSF4 and METTL3 but not of MORC which only resulted in a conventional upregulation of the initially repressed ***TGME49_206400*** gene. On the bottom is a histogram representation of Epinano differential error z-score between METTL3 UT and IAA conditions. (**bottom left panel**) FLAIR derived splicing read-through isoforms into the ***TGME49_250220*** locus are displayed, as well as the differential splicing outcomes on the respective exons, resulting in chimeric RNAs. <u>Notable observations</u>: the nanopore data was mapped against an unannotated gene and is not detected by the ***illumina***-seq, as it was mapped against the predicted transcriptome and not genomic DNA, contrary to DRS which is aligned on genomic DNA.

Supplementary file 4

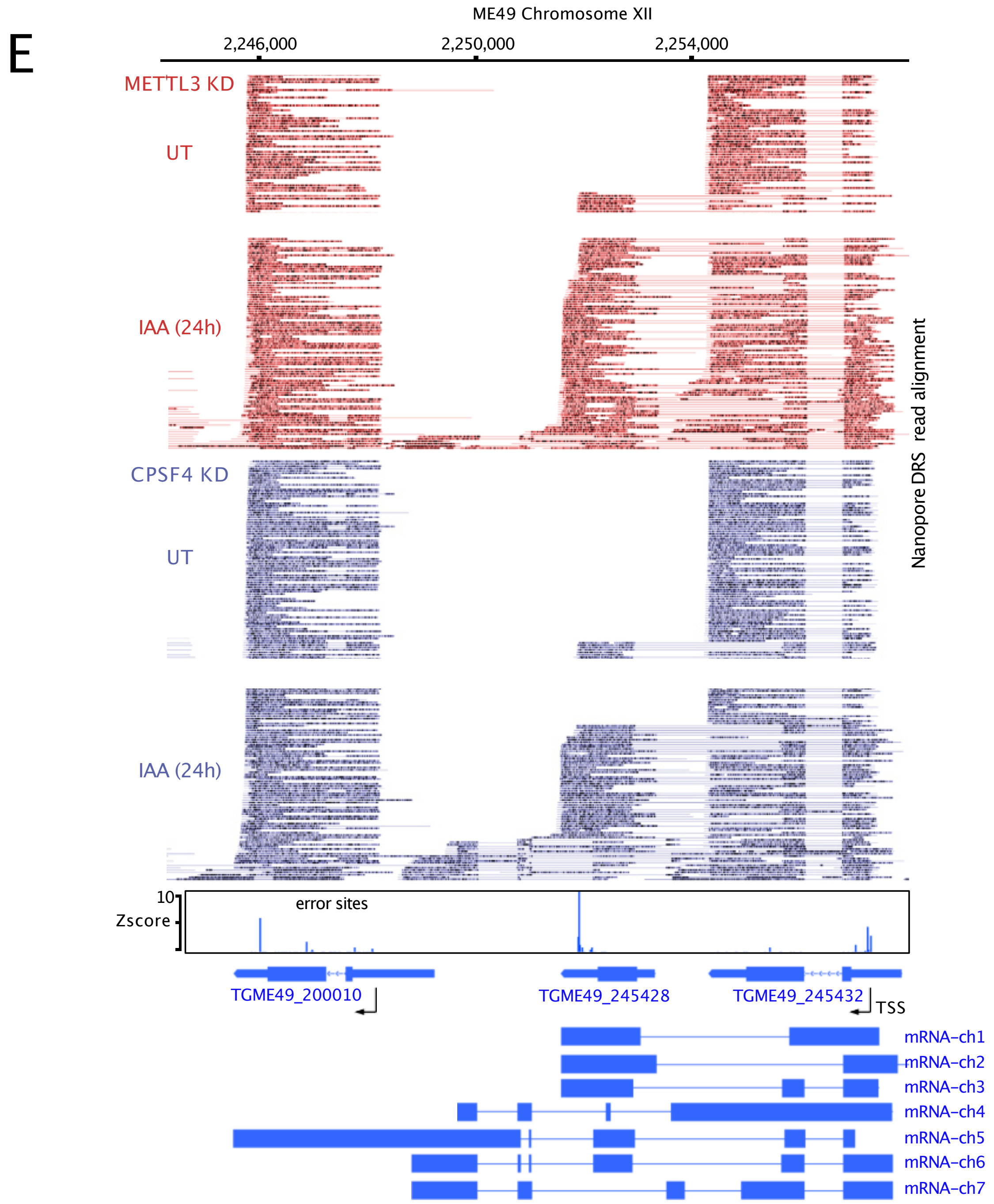

Read-through events and chimeric RNAs detected near TGME49_245432 locus following CPSF4 and METTL3 post-translational knock-down. (**top panel**) A representative analysis of the read-through from ***TGME49_245432*** into the ***TGME49_245428*** locus and beyond. Aligned DRS reads of RNAs extracted before and after the IAA-dependent knock-down of METTL3 (in red) and of CPSF4 (in blue). Exons are shown with colored thick bars while introns and soft clips with thin lines, no strand specific colors are used. ***Epinano*** differential error Z-score between METTL3 UT and IAA conditions are shown as blue histograms on the bottom. (**bottom panel**) FLAIR derived read-through and splicing isoforms of the ***TGME49_245432*** into the ***TGME49_245428*** and ***TGME49_200010 loci*** are displayed, as well as the differential splicing outcomes on the respective exons, resulting in chimeric RNAs.

Supplementary file 4

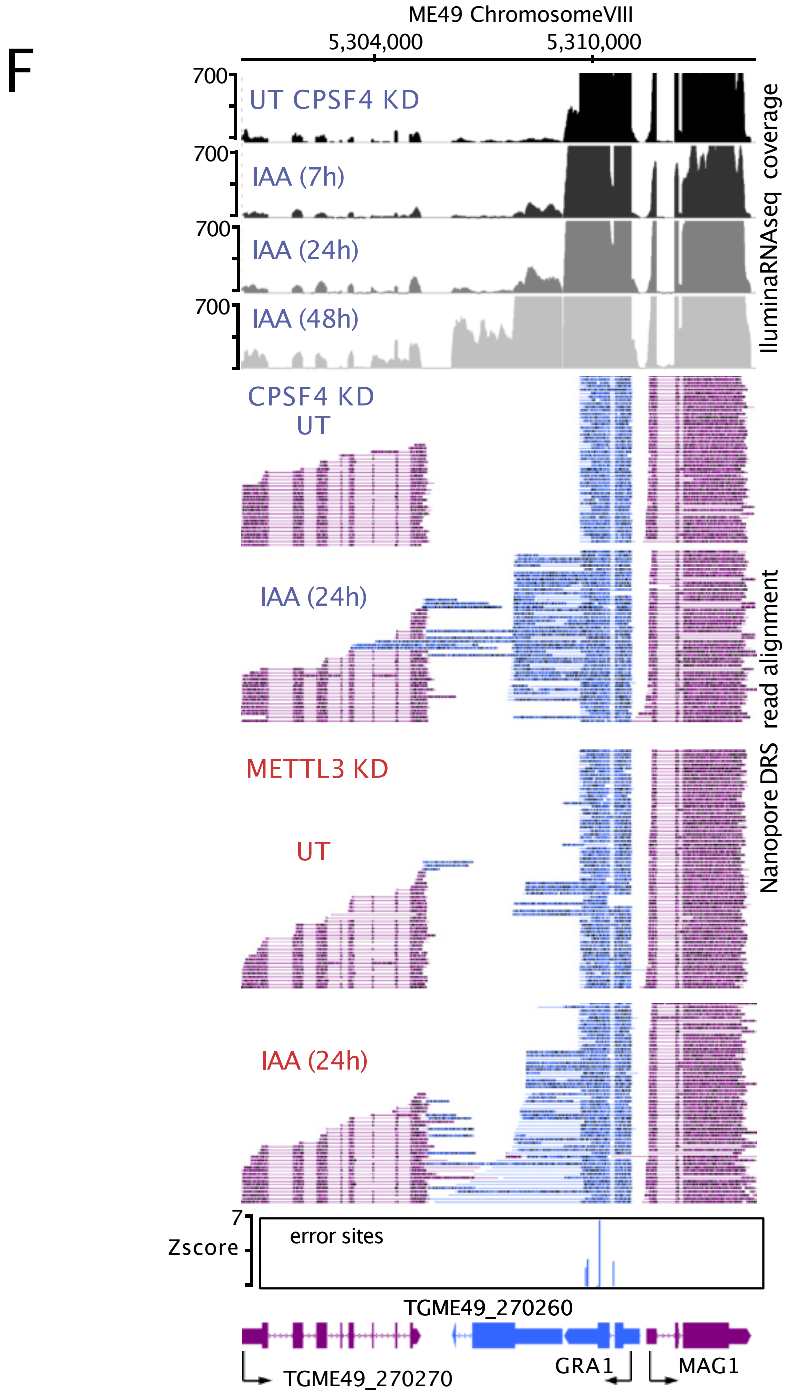

Read-through events and chimeric RNAs detected near GRA1-MAG1 locus following CPSF4 and METTL3 post-translational knock-down. A representative analysis of the read-through from the GRA1 into the ***TGME49_270260 locus***. On top are displayed the ***illumina***-RNA-seq data before and after the IAA-dependent KD of CPSF4, at different times. The y-axis represents the RPKM values. Below are DRS single molecule alignments of RNAs extracted before and after the IAA-dependent knock-down of METTL3 and of CPSF4. Positive strand reads are colored in magenta while negative strand reads are colored in blue. Exons are shown with colored thick bars and introns or soft clips with thin lines. ***Epinano*** differential error sites (Z-score >5) are shown in a histogram fashion.

Supplementary file 4

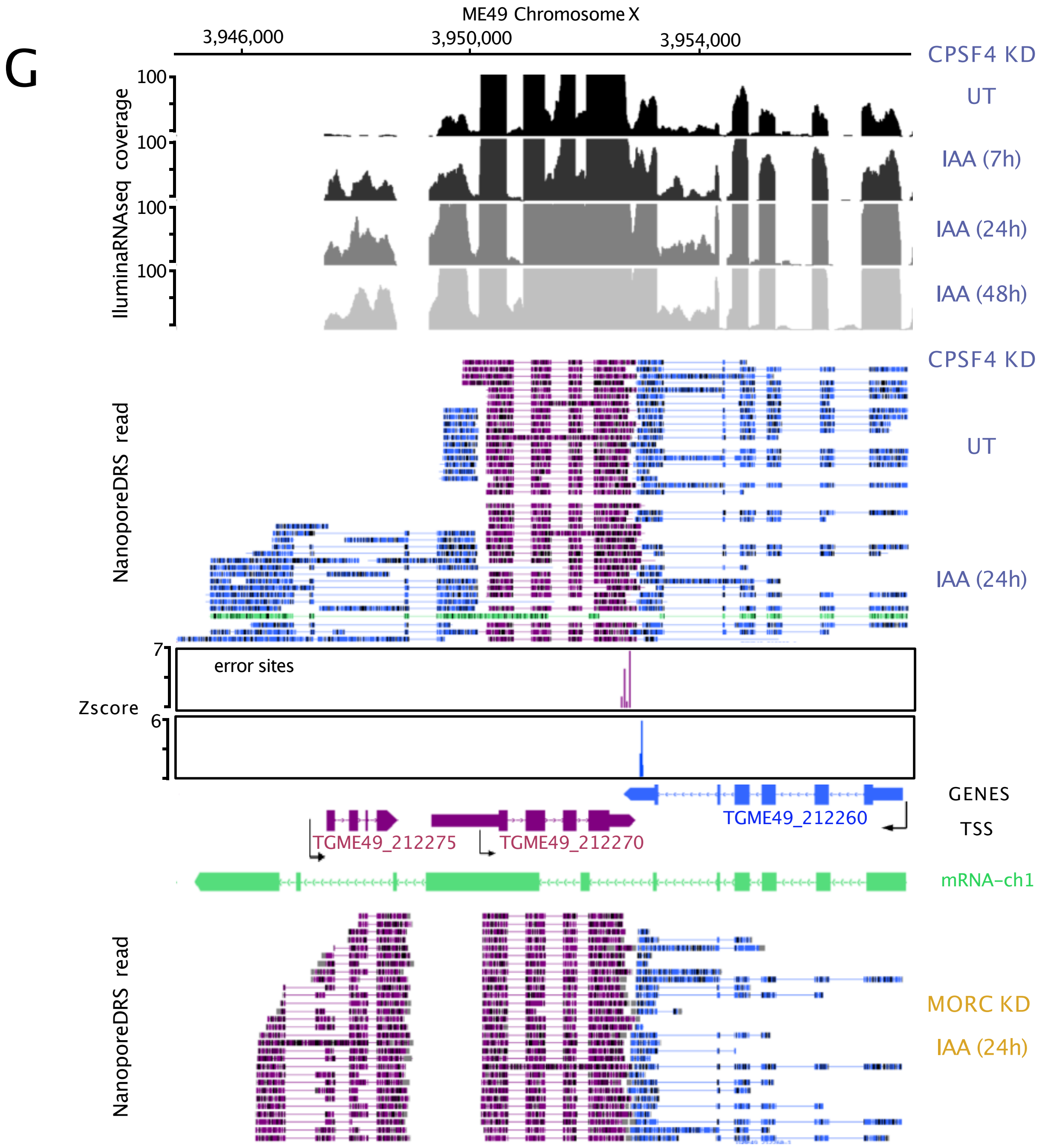

Read-through events and chimeric RNAs detected near TGME49_212260 locus following CPSF4 and METTL3 post-translational knock-down. A representative analysis of the read-through from the TGME49_212260 into the ***TGME49_212270*** and ***TGM49_21227*** genes. On top are displayed the ***illumina***-RNA-seq data before and after the IAA-dependent KD of CPSF4, at different times. The y-axis represents the RPKM values. Below DRS aligned reads from RNAs extracted before and after the IAA-dependent knock-down of CPSF4 and of MORC. Exons are shown with colored thick bars and introns in thinner lines. Positive strand reads are colored in magenta while negative strand reads are colored in blue. ***Epinano*** differential error sites (Z-score >5) are indicated in the same strand specific coloring scheme. An extreme example of negative sense readthrough (RNA chimera 1, shown in green) is displayed following the KD of both CPSF4. Below is a MORC KD which only resulted in a conventional upregulation of the initially repressed ***TGM49_212275*** gene, with no transcripts fusions.

Supplementary file 4

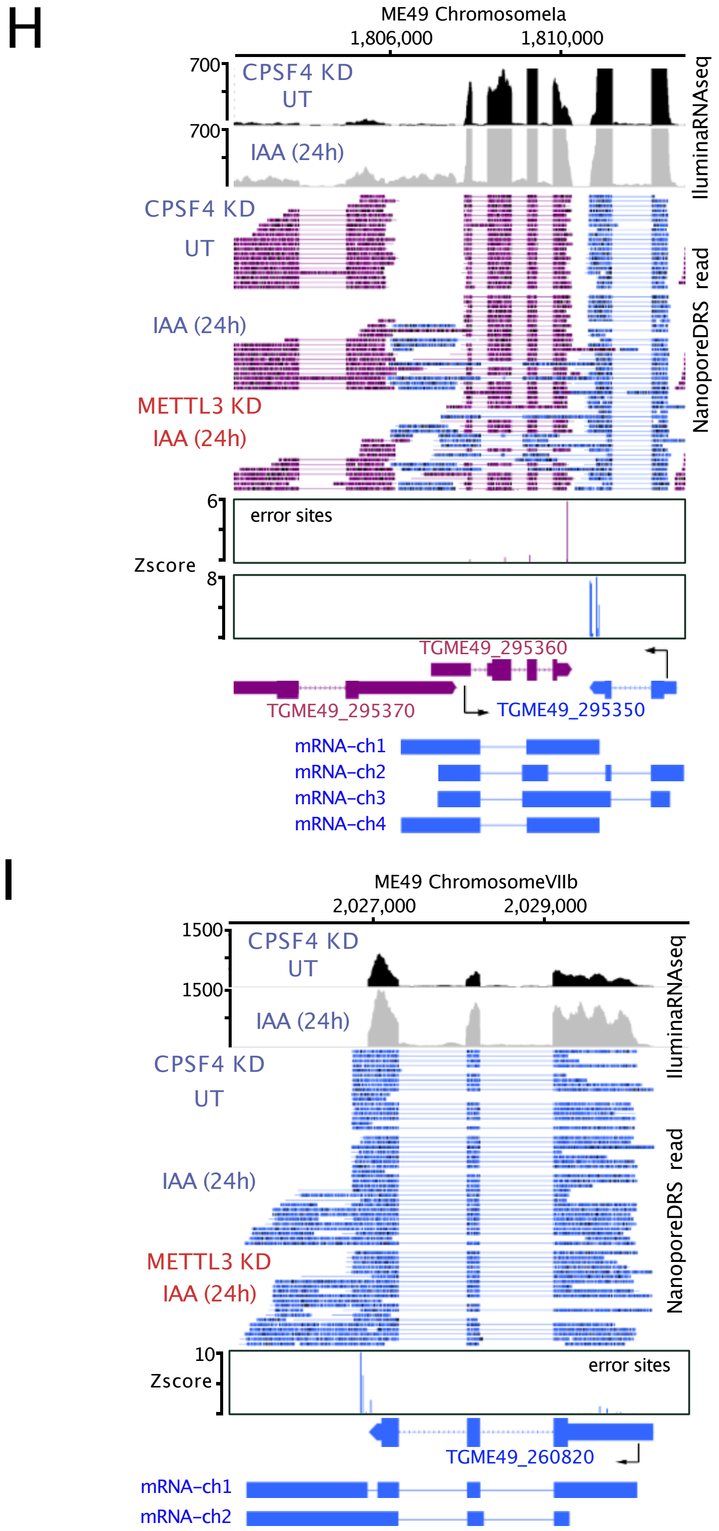

Read-through events and chimeric RNAs detected on T. gondii chromosomes Ia and VIIb following CPSF4 and METTL3 post-translational knock-down. (**H**) A representative analysis of the read-through from the ***TGME49_295350*** into the ***TGME49_295360 locus***. On top are displayed the ***illumina***-RNA-seq data before and after the IAA-dependent KD of CPSF4, at 24 hours. The y-axis represents the RPKM values. Below DRS mapped reads of RNAs extracted before and after the IAA-dependent knock-down of METTL3 and of CPSF4. Positive strand reads are colored in magenta while negative strand reads are colored in blue. Exons are shown with colored thick bars and introns with thinner lines. ***Epinano*** derived differential error sites (Z-score >5) are indicated with the same strand specific coloring rules. The read-through into the ***TGME49_295360 locus*** and beyond is displayed, as well as the differential splicing outcomes on the respective exons, resulting in chimeric RNAs. (**I**) A representative analysis of the read-through at the ***TGME49_260820*** locus with the same description rules as in (**H**).

Supplementary file 4
