# Supplementary material for: A plant-like mechanism coupling m6A reading to polyadenylation safeguards transcriptome integrity and developmental gene partitioning in Toxoplasma
Source: eLife. 2021 Jul 15;10:e68312. doi: 10.7554/eLife.68312 (PMC8313237; doi:10.7554/eLife.68312)
Supplement: Supplementary file 5. [file elife-68312-supp5.pdf]

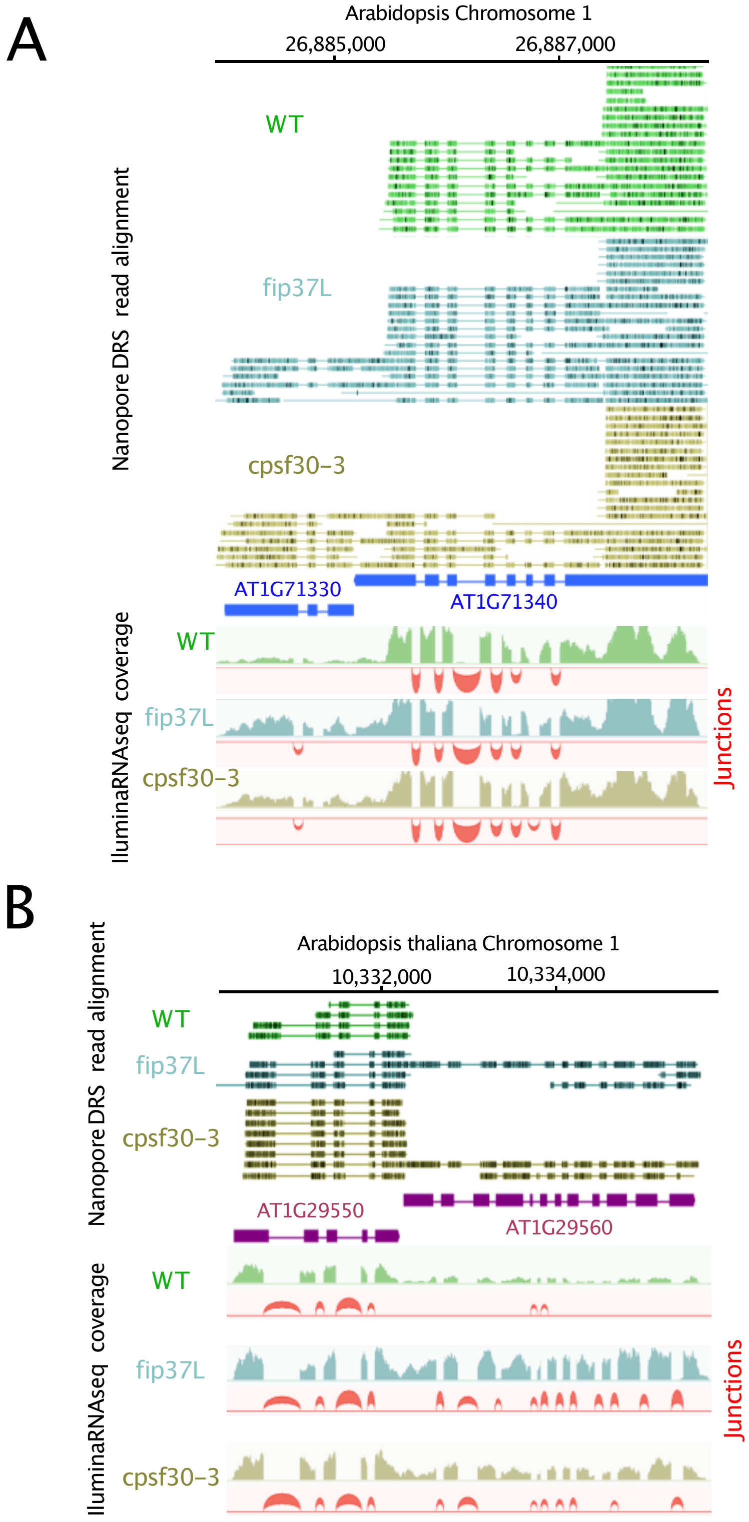

Read-through events and chimeric RNAs detected on A. thaliana chromosome 1, 2 and 5 in the context of fip37L and cpsf30-3 mutants. **(A)** Aligned reads or density profiles from both DRS (top) and ***illumina*** RNA-seq data (bottom) of the ***AT1G71330-AT1G71340*** loci displaying a read-through when the RNAs are extracted from plants harboring either the fip37L mutation, or the CPSF30-3 mutation which specifically abrogates the CPSF30L mRNA production (see S1D Fig). The ***illumina*** RNA-seq data are represented by sashimi plots showing the differential splicing outcomes on the introns, in the backgrounds of these mutations. **(B)** Aligned reads or density profiles from both Nanopore RNA-sequencing (top) and illumina RNA-seq data (bottom) of the ***AT1G29550- AT1G29560*** loci. Similar description as in (**A**).

Supplementary file 5

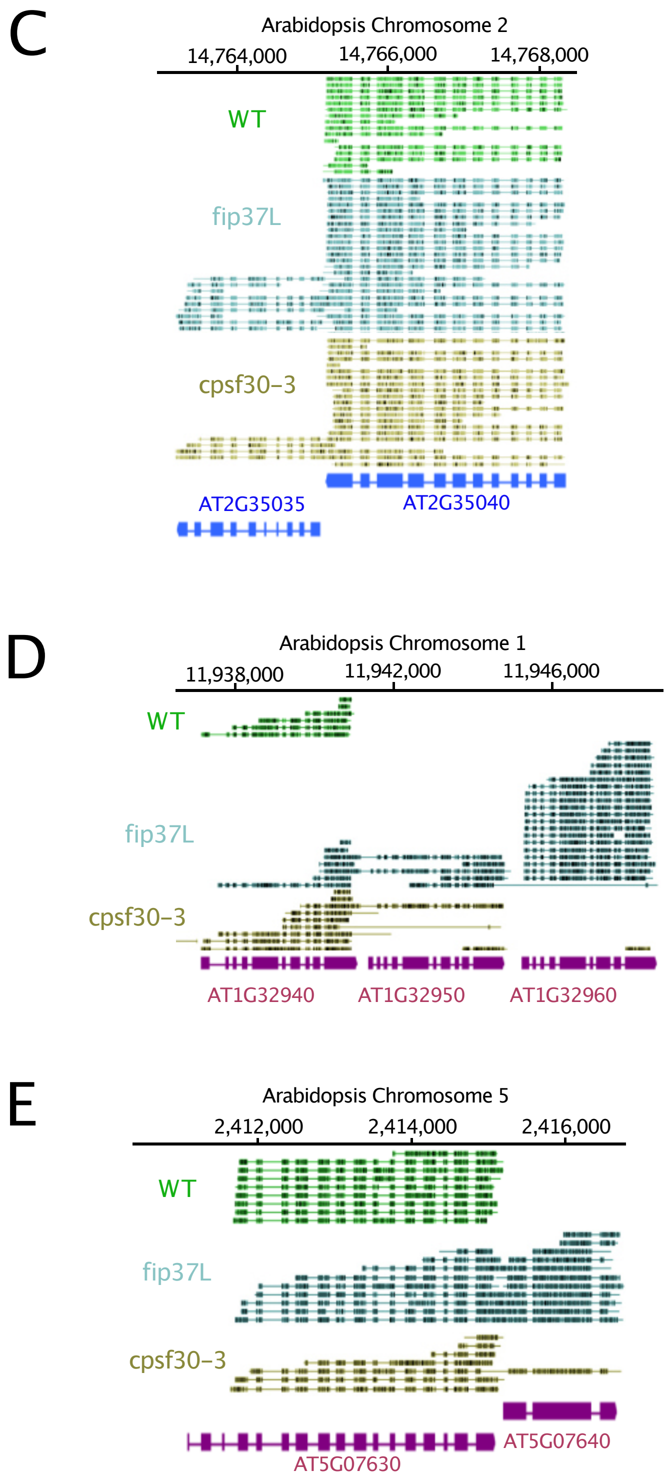

Read-through events and chimeric RNAs detected on A. thaliana chromosome 1, 2 and 5 in the context of fip37L and cpsf30-3 mutants. **(C)** Aligned reads or density profiles from both Nanopore RNA-sequencing (top) and ***illumina*** RNA-seq data (bottom) of the ***AT2G35035-AT2G35040 loci***. Similar description as in (**A**). **(D)** Aligned reads or density profiles from both Nanopore RNA-sequencing (top) and illumina RNA-seq data (bottom) of the ***AT1G32940-AT1G32950 loci***. Similar description as in (**A**). **(E)** Aligned reads or density profiles from both Nanopore RNA-sequencing (top) and illumina RNA-seq data (bottom) of the ***AT5G07630-AT5G07640*** loci. Similar description as in (**A**).

Supplementary file 5
