# Supplementary material for: A plant-like mechanism coupling m6A reading to polyadenylation safeguards transcriptome integrity and developmental gene partitioning in Toxoplasma
Source: eLife. 2021 Jul 15;10:e68312. doi: 10.7554/eLife.68312 (PMC8313237; doi:10.7554/eLife.68312)
Supplement: Transparent reporting form [file elife-68312-transrepform.docx]

***eLife’s* transparent reporting form**

We encourage authors to provide detailed information *within their submission* to facilitate the interpretation and replication of experiments. Authors can upload supporting documentation to indicate the use of appropriate reporting guidelines for health-related research (see [EQUATOR Network](http://www.equator-network.org/%20)), life science research (see the [BioSharing Information Resource](https://biosharing.org/)), or the [ARRIVE guidelines](http://www.plosbiology.org/article/info:doi/10.1371/journal.pbio.1000412) for reporting work involving animal research. Where applicable, authors should refer to any relevant reporting standards documents in this form.

If you have any questions, please consult our Journal Policies and/or contact us: [editorial@elifesciences.org](mailto:editorial@elifesciences.org).

**Sample-size estimation**

- You should state whether an appropriate sample size was computed when the study was being designed
- You should state the statistical method of sample size computation and any required assumptions
- If no explicit power analysis was used, you should describe how you decided what sample (replicate) size (number) to use

Please outline where this information can be found within the submission (e.g., sections or figure legends), or explain why this information doesn’t apply to your submission:

No explicit power analysis was used as the results were relatively consistent between samples.

**Replicates**

- You should report how often each experiment was performed
- You should include a definition of biological versus technical replication
- The data obtained should be provided and sufficient information should be provided to indicate the number of independent biological and/or technical replicates
- If you encountered any outliers, you should describe how these were handled
- Criteria for exclusion/inclusion of data should be clearly stated
- High-throughput sequence data should be uploaded before submission, with a private link for reviewers provided (these are available from both GEO and ArrayExpress)

Please outline where this information can be found within the submission (e.g., sections or figure legends), or explain why this information doesn’t apply to your submission:

In the “materials and methods” section, we describe the number of replicates, and the criteria used to exclude samples, if applicable.

*Immunofluorescence assays* were performed at least in duplicate (technical replication) and at least in two independent experiments (biological replicates).

**Statistical reporting**

- Statistical analysis methods should be described and justified
- Raw data should be presented in figures whenever informative to do so (typically when N per group is less than 10)
- For each experiment, you should identify the statistical tests used, exact values of N, definitions of center, methods of multiple test correction, and dispersion and precision measures (e.g., mean, median, SD, SEM, confidence intervals; and, for the major substantive results, a measure of effect size (e.g., Pearson's r, Cohen's d)
- Report exact p-values wherever possible alongside the summary statistics and 95% confidence intervals. These should be reported for all key questions and not only when the p-value is less than 0.05.

Please outline where this information can be found within the submission (e.g., sections or figure legends), or explain why this information doesn’t apply to your submission:

Detailed information with regards to all statistical analyses performed can be found in the legends for each figure and also in the results text.

(For large datasets, or papers with a very large number of statistical tests, you may upload a single table file with tests, Ns, etc., with reference to sections in the manuscript.)

**Group allocation**

- Indicate how samples were allocated into experimental groups (in the case of clinical studies, please specify allocation to treatment method); if randomization was used, please also state if restricted randomization was applied
- Indicate if masking was used during group allocation, data collection and/or data analysis

Please outline where this information can be found within the submission (e.g., sections or figure legends), or explain why this information doesn’t apply to your submission:

No group allocation was applied.

**Additional data files (“source data”)**

- We encourage you to upload relevant additional data files, such as numerical data that are represented as a graph in a figure, or as a summary table
- Where provided, these should be in the most useful format, and they can be uploaded as “Source data” files linked to a main figure or table
- Include model definition files including the full list of parameters used
- Include code used for data analysis (e.g., R, MatLab)
- Avoid stating that data files are “available upon request”

Please indicate the figures or tables for which source data files have been provided:

The Nanopore RNAseq data have been deposited in NCBI’s SRA data PRJNA705300. To review go :

<https://dataview.ncbi.nlm.nih.gov/object/PRJNA705300?reviewer=eif191tvb5jqrbfk2bvfv9qdt2>

**Supplementary file 1.** Table: Mass spectrometry quantitation of proteins from T. gondii CPSF and m6A writer complexes. Mass spectrometry-based proteomic analysis of Flag elution identified CPSF1, WDR33, Fip1, PAP, METTL3, METTL14 and WTAP and their partners. The identities of the proteins (accession number on ToxoDB, gene name and description) are indicated. For each protein, MaxQuant reports an intensity-based absolute quantification (iBAQ) value, a measure of protein abundance.

The MS proteomics data have been deposited to the ProteomeXchange Consortium through the PRIDE partner repository with the dataset identifier PXD024326.

Access to reviewers:

<https://www.ebi.ac.uk/pride/login>

Username: reviewer_pxd024326@ebi.ac.uk

Password: xCZg9WpK

**Supplementary file 2.** Table: Crystallography data statistics.

**Supplementary file 3**. Table: Analysis of Illumina high-throughput RNA-Seq data of the CPSF4 KD parasite line of T. gondii, related to S5 Fig. RNA-Seq report, Raw counts of T. gondii transcripts generated by feature Counts (Subread), Normalized T. gondii transcripts (TPM), Differential expression analysis (iDEP.92), data used in volcano plot (48h vs UT). The Illumina RNAseq and gene-wise quantifications have been deposited in NCBI’s Gene Expression Omnibus and are accessible through GEO Serie accession number GSE168155.

To review GEO accession GSE168155:

Go to <https://www.ncbi.nlm.nih.gov/geo/query/acc.cgi?acc=GSE168155>

Enter token czsjqeysxvutrsx into the box

**Supplementary file 4.** Supplementary figure showing Read-through events and chimeric RNAs detected on *T. gondii* chromosomes following CPSF4 and METTL3 post-translational knock-down.

**Supplementary file 5.** Supplementary figure showing Read-through events and chimeric RNAs detected on *A. thaliana* chromosomes in the context of *fip37L* and *cpsf30-3* mutants.

**Supplementary file 6.** Table: T. gondii Strains, Vectors and Primers. List of T. gondii parasite strains and transgenic lines as well as plasmids used in this work. Primers and DNA construct used in this work are also charted in the table.

**Supplementary file 7** : Full wwPDB X-ray Structure Validation Report of Crystal structure of Toxoplasma CPSF4-YTH domain in apo form (PDB ID : 7NG2)

**Supplementary file 8** : Full wwPDB X-ray Structure Validation Report of Crystal structure of Toxoplasma CPSF4-YTH domain bound to m6A (PDB ID : 7NH2)

**Supplementary file 9** : Full wwPDB X-ray Structure Validation Report of Crystal structure of the Toxoplasma CPSF4 YTH-domain in complex with a 7 mer m6A-modified RNA (PDB ID : 7NJC)
